# Digital Resilience Through Training Protocols: Learning To Identify Fake News On Social Media

**DOI:** 10.1007/s10796-021-10240-7

**Published:** 2022-01-19

**Authors:** Lisa Soetekouw, Spyros Angelopoulos

**Affiliations:** 1 University of Amsterdam, Amsterdam, The Netherlands; 2 Durham University Business School, Durham University, Durham, UK

**Keywords:** Fake news, Misinformation, Fake news detection, Social media, Scepticism

## Abstract

We explore whether training protocols can enhance the ability of social media users to detect fake news, by conducting an online experiment (*N* = 417) to analyse the effect of such a training protocol, while considering the role of scepticism, age, and level of education. Our findings show a significant relationship between the training protocol and the ability of social media users to detect fake news, suggesting that the protocol can play a positive role in training social media users to recognize fake news. Moreover, we find a direct positive relationship between age and level of education on the one hand and ability to detect fake news on the other, which has implications for future research. We demonstrate the potential of training protocols in countering the effects of fake news, as a scalable solution that empowers users and addresses concerns about the time-consuming nature of fact-checking.

## Introduction

The problem of fake news and misinformation is currently often discussed, specifically in the context of social media. Whilst the concepts are not new (Tandoc et al., 2018), the way fake news spread through social media has changed the game (Carlson, 2018), and it is not clear where the responsibility lies for countering their spread (Helberger et al., 2018). It becomes, therefore, crucial and simultaneously difficult to clearly define what fake news entails (Berghel, 2017). However, numerous examples can be given where fake news allegedly had political consequences (Timmer, 2016), and the increasing spread of misinformation affects our trust in the media (Lazer et al., 2018; Vaccari & Chadwick, 2020). Several social media organizations, including Facebook and Twitter, have started deleting fake news and the profiles of users spreading them. The most notable example is the ban of the former President Trump from many social media (Denham, 2021). The role of social media platforms in society has often been debated. Facebook, for example, is not a traditional news outlet, but arguably is the largest news publisher currently existing (Carlson, 2018). Moreover, social media platforms have come to play a big part in our daily lives and it has been reasoned this comes with certain accountability (Helberger et al., 2018).

The impact of fake news has recently grown (Vishwanath, 2015), especially since the 2016 US elections, when a heated discussion started regarding the impact of fake news and the role that social media play in it (Allcott & Gentzkow, 2017). Moreover, during the COVID-19 pandemic the debate about the spread of misinformation has centred around health-related consequences. Fake news have been reported to receive more exposure than stories from mainstream sources (Parra et al., 2021; Zhou et al., 2019), and recent research highlights the need for the public to receive information from health authorities rather than social media (Kim & Kim, 2020). However, with a large portion of the population relying on social media as their main information source (Lazer et al., 2018), the challenge of educating the public about the impact of misinformation and providing them with tools to recognize fake news remains. Additionally, fake news has led to a decrease in trust in authorities and mainstream news outlets (Vaccari & Chadwick, 2020), and prior research shows that the extent to which one believes fake news is related to several factors, including one’s political views and education (Halpern et al., 2019). Arguably, thus, the calls to rely on information and news shared by authorities may not effectively convince some groups.

Concurrently, the rise of deepfake videos seems to have given a new dimension to the existing debate. Once again, the technology behind deepfakes is not new, but the quality of these videos has been increasing and the technology is easily accessible (Kietzmann et al., 2020). Subsequently, the widespread deployment of this technology has given rise to concern that, in the near future, it will be even more difficult to distinguish what is fake and what is real (Fallis, 2021; Kietzmann et al., 2020). Furthermore, the impact of deepfakes seems far-reaching because it has the potential to create false memories (Liv & Greenbaum, 2020). Several solutions to the fake news problem have been considered. The potential use of Artificial Intelligence (AI) plays a large role in these discussions (Kreft & Fydrych, 2018), but the role that experts may be able to play also receives more attention (Clayton et al., 2020). We aim to provide an analysis of how the public can be trained to recognize fake news, and what practices are effective for this purpose. The research question that we are tackling here, is:

### To what extent can social media users be trained to detect fake news using training protocols?

To answer the research question, we incorporate an experiment-based approach, designed to test how effective a protocol is in training the public to detect fake news. This protocol is based on prior literature and experts’ knowledge and can serve as a measure to counter the impact of fake news, possibly in addition to machine-based measures that are currently being developed. As the potential of such training mechanisms has not been studied yet, our work provides a starting point for further research into what types of protocols work best.

The remainder of this paper is structured as follows. The next section provides an overview of the existing literature. After this, the promises of training the public are discussed and the foundation for the development of the training protocol is laid. The following section provides a description of the methodology, followed by the results of our study. Next, these results are discussed, and their implications are also considered. Finally, a brief conclusion is provided along with limitations, as well as an agenda for future research on the topic.

## Theoretical Background

### Fake News on Social Media

Social media users face a difficulty in distinguishing fake news (Allcott & Gentzkow, 2017; Au et al., 2021; Borges & Gambarato, 2019) and this issue becomes more complex due to the rise of new technologies, such as deepfakes. Social media facilitate the fast spread of fake news (Kreft & Fydrych, 2018), and prior research shows that fake news enjoy higher levels of exposure (Timmer, 2017) due to the involvement of bots (Vosoughi et al., 2018). Moreover, fake news affect the credibility of traditional news outlets (Fallis, 2021), with trust in such outlets measured at an historic low (Lazer et al., 2018). Concurrently, the number of people relying on social media to find news has increased (Rubin et al., 2016). Several studies point towards humans not being good at recognizing fake news (e.g., Bond & DePaulo, 2006; Rubin et al., 2016). Without further training or tools, people score about 54% on tasks in which they need to distinguish truth and deception—only slightly better than chance. However, a number of questions regarding what can be done to counter the effect of fake news remain to be addressed (Au et al., 2021), and scientific evidence for the use of certain tools is limited (Paredes et al., 2021).

New technologies and recent events, such as the US elections and the Brexit referendum, seem to attract more interest in the topic (Zhou et al., 2019). In fact, fake news lead to an increase in (political) polarization (Riedel et al., 2017), and is frequently described as a single, straightforward phenomenon. However, a recent study points towards the need to consider it as a two-dimensional phenomenon (Egelhofer & Lecheler, 2019), suggesting to distinguish between fake news as a genre and fake news as a label. The former points towards the intentional creation of fake news, while the latter refers to the use of the term ‘fake news’ to invalidate the media. We focus on fake news as a genre, but when considering literature focusing on this dimension, definitions of fake news still contain many elements. A common element in these definitions is that fake news refers to messages, of any kind, containing false information (Bakir & McStay, 2018; Lazer et al., 2018). Moreover, while fake news come in many forms, many authors see the imitation or mimicking of real news messages as an important aspect (Lazer et al., 2018). Third, we often see fake news described as not verifiable through facts and figures (Gimpel et al., 2020). We follow, the definitions of Lazer et al. (2018), who define the concepts as “fabricated information that mimics news media content in form but not in organizational process or intent” (Lazer et al., 2018, p. 1094). Misinformation here is simply defined as information that is either false or misleading (Lazer et al., 2018).

The debates surrounding these concepts appear to focus on the role of social media and new technologies. One of the most striking technological developments arguably is the rise of deepfakes, which are fake videos that are developed using AI that allows them to seem like someone says or does something they actually never did (Dobber et al., 2021). Deepfakes are feared to have impact in times of political elections as continuously improving an easily accessible technology makes it easier to fabricate such videos and more difficult to distinguish them from real ones (Fallis, 2021). Specifically, it is important to consider how deepfakes differ from photoshopped images. While photoshopped images mislead in terms of what we see, deepfakes also affect what we hear (Dobber et al., 2021). The increasing extent to which deepfakes are perceived as realistic or real has impact on society. Prior research has evaluated the impact of deepfakes in the context of political microtargeting, an increasingly employed technique in which information is gathered on individuals to enable targeted information during, for example, electoral periods (Borgesius et al., 2018). Previous research has emphasized that deepfakes have the potential to affect attitudes and, especially due to the rapid developments in terms of quality and ease of fabrication, should be expected to have more impact in the future (Dobber et al., 2021).

Social media plays a large role in the spread of misinformation, both in the form of deepfakes and in other forms (Borges & Gambarato, 2019). While traditional media is characterized by a relative balance in the news that is presented, the goal of large social media corporations is to retain their users (Carlson, 2018). To achieve this, the content presented to users is tailored to their preferences through algorithms. While algorithms might seem neutral due to their data-driven nature, humans are involved in their training and biases inevitably are built into their design (Gillespie, 2014). Moreover, the inner processes of algorithms are unclear or difficult to understand (Carlson, 2018). As a result, filter bubbles and echo chambers are created due to increasing exposure to personalised content (Borges & Gambarato, 2019), which can lead to the reinforcements of existing beliefs and to intellectual isolation. Homogeneity in the content users are exposed to leads to polarisation of opinions, giving way to the growth of fake news (Kreft & Fydrych, 2018). Such homogeneous content and polarised opinions lead to lower acceptance of opposing views and novel information (Lazer et al., 2018). Prior research confirms that people inherently are more likely to believe news that fits their existing beliefs (Hameleers & van der Meer, 2020). Fake news anticipates on this, and shows users what they want to see (Kreft & Fydrych, 2018). This suggests that fake news is more likely to be perceived to be true by those whose prior beliefs match the content provided. Moreover, the public may not be deceived directly by deepfakes, but that it does lead to feelings of uncertainty (Vaccari & Chadwick, 2020). This uncertainty, in turn, may lead to a decrease in trust in traditional news outlets. Moreover, such deepfakes affect attitudes regarding politicians (Dobber et al., 2021), an effect that can be enhanced further by microtargeting practices (Borgesius et al., 2018). Moreover, people are said to be vulnerable to fake news, and even those who do not mean to often participate in sharing fake news (Zhou et al., 2019).

### Countering Fake News

In addition, social media allows for quick and easy sharing of large volumes of content, which adds to the challenges of detecting and countering fake news (Zhang & Ghorbani, 2020). On top of this, the way in which news is presented has changed. An often used term to describe this is the ‘tabloitization of news’ (Rowe, 2011), referring to how the speed at which news is delivered is considered more important and revenues from advertisements play a large role. As news outlets want to ensure readers click on their articles, such focus on speed may have consequences for the extent to which articles are fact-checked, which may in turn blur the lines between facts and fiction or unverified information. The increase of such ‘clickbait news’ has often been connected to the developments regarding misinformation. Considering the impact of fake news, there have been several attempts to counter them. Fake news come in various forms, making detection difficult (Zhou et al., 2019). Developing accurate measures is challenging, due to the above-mentioned large volumes of fake news shared on social media (Zhang & Ghorbani, 2020), but the fact that fake news consists of many different, complex aspects adds to this as well (Ruchansky et al., 2017). Lazer et al. (2018) identify two categories of measures, one of which refers to detection and intervention on platforms, the other focusing on empowering individuals. The former is about detection and intervention on platforms and involves the employment of algorithms. There exists a considerable body of literature focusing on how data mining can be employed to detect fake news of social media (Ciampaglia et al., 2015; Conroy et al., 2015; Shu et al., 2017). Algorithms and AI simultaneously enable the rise and spread of fake news and help counter it (Kreft & Fydrych, 2018). The way social networking sites, such as Facebook, employ their algorithm to enhance consumer engagement, should also be employable for ensuring users are exposed to quality content (Lazer et al., 2018). An example of this would be exposing users to diverse political content, rather than merely content confirming their existing beliefs. This could in turn reduce the effect of echo chambers, a phenomenon caused by and reinforcing the polarized political opinions (Borges & Gambarato, 2019).

The second category addresses the potential of empowering individuals. There have been initiatives to counter the effects of fake news by training social media users. For instance, Facebook released a tutorial with tips on how to recognize fake news (Brady et al., 2017). Moreover, efforts to uncover the truth behind fake news stories have been made by fact-checkers (Hameleers & van der Meer, 2020). Using expert knowledge is not a new approach to countering fake news. It has, in fact been deployed for several decades (Fridkin et al., 2015). However, fact-checking conducted by experts seems to have risen as a response to growing misinformation revolving around politics (Fridkin et al., 2015). Recent studies show the potential of employing such experts (Clayton et al., 2020). Fact-checkers can potentially reduce polarization and help dealing with partisan identities (Hameleers & van der Meer, 2020) and that they affect people’s evaluation of the accuracy of political messages (Fridkin et al., 2015).

It is important to consider the limitations of deploying fact-checkers to counter the effects of fake news, as fact-checkers are only effective when correcting information that fits the prior beliefs of the person exposed to it (Hameleers & van der Meer, 2020). This means that fact-checking efforts bring those with polarized opinions on either side closer together, having potential to bridge the gap. Although the so-called backfire effect, explaining how presenting factual information to counter fake news will only lead to a stronger belief in the presented misinformation (Nyhan & Reifler, 2010) has raised concern, recent research emphasizes that evidence for such an effect is weaker than initially thought (Wood & Porter, 2019). However, merely employing fact-checking is not enough to deal with the fast-moving developments in the area of fake news (Ciampaglia et al., 2015).

Although these two approaches are often discussed distinctly, there exists literature arguing for a more hybrid approach as well. It is, for instance, argued that machine-based and human-based approaches should not be seen as mutually exclusive (Okoro et al., 2018). Moreover, the technologies that are currently used and developed are time-consuming and the fast-moving developments add to their complexity. Studies thus argue for the need to equip people with the right tools and knowledge to detect fake news (Zhang & Ghorbani, 2020). Moreover, to develop effective measures, joint effort of expert from all kinds of disciplines is necessary (Zhou et al., 2019). While the potential impact of fact-checkers has been considered and recent studies point towards the potential of fact-checking efforts in reducing the effects of fake news, the evidence for such efforts is limited (Lazer et al., 2018), and questions as to what type of protocols work and how they can be deployed remain, which means it is not clear how we can implement such methods and which factors are most important to consider.

## Hypotheses Development

Part of the intervention methods currently employed consists of automated approaches to restrict the extent to which fake news can be spread. Prior studies have recognized that tackling the problem from the demand-side can be beneficial and that more attention should be given to intervention rather than detection (Sharma et al., 2019). For example, research has provided evidence for the positive impact of showing related controversial articles alongside fake news (Gimpel et al., 2020). We define fake news detection as “the correct decision of an individual that information is false” (Gimpel et al., 2020, p. 6065). To improve users’ ability to detect fake news and misinformation, it is important to take news media literacy into account. News media literacy refers to the extent to which users are able to critically evaluate the news messages they encounter (Ashley et al., 2013). Although many people think they are able to separate fake news from real news, they often do not perform well when presented with a task (Auberry, 2018). Tools that are currently being developed for detecting fake news, for instance based on AI, do not help users to develop skills to individually assess news messages. However, it has been reported that developing tools with the purpose of training individuals has potential. Auberry (2018), for instance, found that such instruments can help the public in evaluating whether a source is credible and to verify facts. Moreover, previous research showed that such education of the public plays a large role in countering the effects of fake news (Dumitru, 2020). People are, in general, not good at detecting deception in any context. They are inclined to believe what they see (Conroy et al., 2015) and research shows that social media users often do not possess the right skills to critically asses news presented to them (Grace & Hone, 2019). There are many ways in which tools can be shaped to educate social media users, including showing related (controversial) articles, warning, and explanations alongside news messages. Prior studies give a better idea as to what measures are most effective for this purpose. Kirchner and Reuter (2020), for instance, show that social media users prefer transparent intervention methods, in part because this enables them to draw their own, informed conclusions. Moreover, social media users prefer warnings and explanations over other methods. Combining such warning with how peers evaluate the messages, for instance by showing how many friends think the message is fake, worked as well.

Other research gives further insight into how these tools can be designed. For instance, when considering tagging news messages to inform and educate social media users, “rated false” tags on Facebook work better than a “disputed” tag (Clayton et al., 2020). An often-voiced concern with this approach is the challenge of flagging all articles. Clayton et al. (2020) found that even if a fake news article is not flagged by either the “rated false” or the “disputed” flag, it does not necessarily mean the article is perceived to be more accurate. General warnings without a link to specific messages have a positive impact as well, but it should be noted that the effect is smaller. Moreover, such warnings potentially have a detrimental effect on how actual news messages are perceived.

**Fig. 1:**
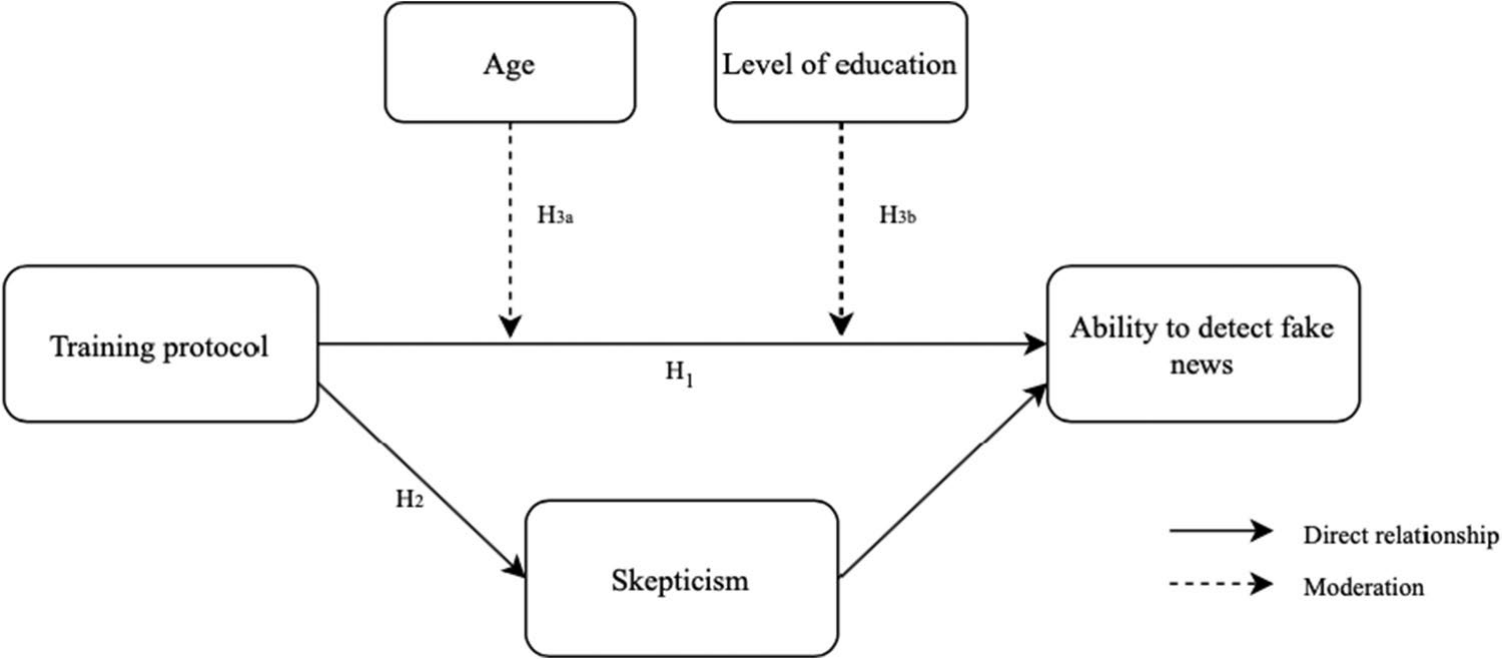
Conceptual Framework

In this paper, we investigate the use of training protocols enhance users’ ability to detect fake news. The focus, thus, lies on the direct relationship between the training protocol and individual’s ability to distinguish between real and fake news messages. Furthermore, our study addresses whether scepticism—potentially enhanced by being exposed to the protocol—explains part of this direct relationship. Finally, the role demographic factors may play are taken into consideration. Figure 1 presents the schematic illustration of these relationships.

### Expert Knowledge

Deploying fact-checkers is often presented as a solution to the fake news problem (Fridkin et al., 2015). However, one of the most important challenges is that the fact-checking process required a lot of time and effort and, therefore, is difficult to scale (Ciampaglia et al., 2015). Moreover, a large number of popular fact-checking websites relies on manual detection (Zhang & Ghorbani, 2020). Many have suggested the employment of technology to take over this detection task. However, such technologies have limitations of their own, for instance because the characteristics of fake news change continuously. Furthermore, the development of such technologies ultimately relies on human efforts (Zhang & Ghorbani, 2020). In response to this, some studies have suggested considering how and to which extent the general audience can participate (Kim & Dennis, 2019). To develop effective measures, joint effort of experts from all kinds of disciplines is necessary (Zhou et al., 2019). This suggests expert fact-checkers can play a valuable role in the development of training mechanisms. We expect, thus, that training protocols based on fact-checkers’ knowledge will have a positive effect on users’ ability to detect fake news. In addition to this, prior research points out that employing fact-checkers can add to the extent to which news outlets are perceived to be trustworthy (Amazeen, 2015). Applying this to the current context, suggests that protocols based on experts’ knowledge may be able to enhance the ability to detect fake news and increase the level of trust in news outlets. Moreover, such training protocols can be seen as transparent intervention methods, which, as mentioned before, are preferred by the public (Kirchner & Reuter, 2020). Based on the discussed body of literature, we expect a positive relationship between the employment of a training protocol based on fact checkers’ expertise and the general public’s ability to recognize fake news:

- ***H1:**** A training protocol based on expert knowledge has positive impact on users’ ability to recognize fake news.*

### Scepticism

However, it is important to consider whether this expected relationship could be (in part) explained by other variables. A closer look at the literature on fake news reveals that the presentation of news in certain ways leads to increase scepticism in evaluation of all news articles, including the ones for which the way of presentation had not been not changed (Kim & Dennis, 2019). Therefore, we expect that the training protocol will lead to higher levels of critical evaluation of all news messages. We refer to such enhanced critical thinking as scepticism, and define it as being inclined to not belief information presented, based on research conducted in relation to advertisement scepticism (Obermiller & Spangenberg, 1998). Although many studies have been conducted to explore scepticism in other areas, for instance in relation to climate change (Poortinga et al., 2011; Tranter & Booth, 2015), the question whether it plays a role in fake news detection is still insufficiently explored. We expect scepticism to explain the positive relationship between the training protocol and users’ ability to detect fake news:

- ***H2a:**** Scepticism has a positive impact on users’ ability to recognize fake news.*
- ***H2b:**** Scepticism mediates the relationship between the training protocol and users’ ability to detect fake news.*

### Demographic Factors

When talking about social media, demographics are often taken into consideration. For instance, age and level of education may affect the extent to which one relies on social media to access news (Sindermann et al., 2020). Although more factors might play a role, we focus on age and level education as prior literature gives most reason to believe these may play a role. Regardless of demographics, certain dynamics around fake news makes users to share them via social media. Users might be intrigued by stories about politicians, or the stories might appeal to emotions. Prior research shows that messages with highly emotional content are more likely to be shared (Harber & Cohen, 2005), which could explain why fake news achieves the above-mentioned levels of exposure. Prior research has considered the impact of different demographic factors as well. A recent study by Rampersad and Althiyabi (2020) suggests that age and education may affect the extent to which users accepts news to be true. In the case of age this means that the older one gets the more likely they are to perceive fake news as true. For the level of education this implies that its increase is associated with lower levels of fake news acceptance. Kim and Kim (2020) also echo the suggestion of Harber and Cohen (2005), reporting higher ability to detect fake news for those with higher levels of education. No prior research has reported a reverse relationship. This suggests that the expected positive relationship between the training protocol and one’s ability to detect fake news might be stronger for those with lower levels of education. It has also been suggested that age negatively affects one’s ability to detect fake news, suggesting the older one gets the less likely they are to believe fake news (Sindermann et al., 2020). This has been recognized in other research as well, in which news media literacy among young people seems limited (Loos & Nijenhuis, 2020). While one might think that tech savvy younger generations might be harder to fool, it seems that online misinformation is hard for them to detect. For instance, the results of a study employing a gaming tool show that older participants are better at detecting fake news (Grace & Hone, 2019).

Moreover, young people are more likely to believe fake news than older people. In the context of COVID-19, for instance, it was shown that older people tend to believe more strongly in fake news (Kim & Kim, 2020). However, it is likely that other factors play a role as well. As mentioned, fake news uses emotion to appeal to people and for older generations, COVID-19 has more potential impact that for younger generations. We, thus, assume older people are better at detecting fake news than younger ones. Consequently, it is likely that the effect of the training protocol is less strong for older generations than for younger ones, simply because those older people are better at detecting these stories to begin with. We are not interested in the direct effects of demographics on one’s ability to detect fake news, but rather on how these factors may moderate the direct relationship between the training protocol and one’s ability to detect fake news. Thus, the following set of hypotheses is developed:

- ***H3a:**** Age weakens the relationship between the protocol and users’ ability to detect fake news*
- ***H3b:**** Level of education weakens the relationship between the protocol and users’ ability to detect fake news.*

Providing users with tools to evaluate news on social media is thus important and, according to Zhang and Ghorbani (2020) such tools can be based on three aspects: i) the creator-based approach, ii) the new content approach, and iii) the social context approach. These three aspects can become the basis for the main tips provided in a training protocol. Such a protocol should also be transparent in its purpose, since transparency is preferred by users (Clayton et al., 2020). When it comes to the three main aspects for such a tool (Zhang and Ghorbani, 2020), the creator-based approach is mainly about the source of an article. Prior research indicates that the way in which sources are presented affects the level of scepticism in the audience (Kim & Dennis, 2019). Based on this, educating the public about how exactly to evaluate sources may help them in detecting fake news. This concerns both the direct source of the article and in-article reporting of sources (Kim & Dennis, 2019). In general, people lack the skills to make accurate judgments (Grace & Hone, 2019). In other words, the public need to be educated in how they can examine the evidence at hand. A simple way to incorporate this creator-based approach into the protocol is by explaining how the audience can evaluate whether a URL is trustworthy. The new content approach goes beyond the source and focuses on the content. For instance, Zhang and Ghorbani (2020) argue that not merely reading the headline, checking supporting resources (e.g., statistics), and scrutinizing the sentiment of the message helps in identifying fake news. The latter should help, because fake news stories often focus on appealing to their audience’s emotions, for instance fears. Finally, the social context-based approach goes beyond the content of the message and looks at its context. For instance, it might help to examine other news stories from the same source and to check whether other outlets report on the story. Although all mentioned aspects might be useful to incorporate in a protocol design to, for instance, be distributed via Facebook, not all aspects can be measured here. Therefore, we focus on selected aspects: i) evaluating the source intuitively; ii) evaluating the source using the website URL; iii) evaluating the content using the headline and availability of supporting resources; and iv) examining the sentiment. This means that the social context approach is hard to analyse in the current research.

## Methodology

We conduct a controlled experiment, which is an approach that has been used before in the context of countering fake news, for instance by Gimpel et al. (2020), who explored the impact of showing related articles to enhance detection of fake news. The experiment design allows for detecting causal relationships, and for collecting a large amount of data using few resources. A between-subject design is executed, where all participants are randomly assigned to either the control group or the intervention. To ensure our results are reliable and generalizable at least 200 participants are needed for the study. After finalizing the data collection, tests were conducted to screen the randomization process and ensure even distribution across groups, for instance in terms of age and gender.

The experiment was conducted over Qualtrics, through a survey consisting of six steps. First, a general introduction is shown to all participants. Second, the participants are assigned to the control condition or the intervention. Third, all participants complete the fake news detection task in which they are asked to assess eight news articles. Fourth, the respondents answer a set of questions to assess their level of scepticism. Fifth, questions regarding demographics are asked, which help us to analyse whether demographics can explain the main relationships and allow to check for even distribution across groups. Finally, all participants are debriefed to inform them about the purpose of the research, and the setup of the experiment. Apart from the experimental part, no distinction is made between the two groups. All participants complete the same tasks and follow-up questions.

After the general introduction, the participants are assigned to the control condition or the experimental conditions. Assignment to these groups happens randomly. As the variable ‘Training protocol’ has two levels—the control condition and the intervention—and no other variables are manipulated, it is a 2 × 1 experiment. In case a participant is assigned to the control condition, they merely receive an instruction for completing the fake news detection task (see Appendix I). If assigned to the experimental condition, the participant receives the same instructions for the task, but also sees the training protocol. This training protocol is developed based on prior research and known fact-checking efforts (see Appendix I). This protocol serves as a simple training mechanism that allows the general public to improve their abilities to assess the accurateness and credibility of news messages.

After reading the instructions, all participants regardless of their group, were asked to complete the same task. News stories from different news outlets were selected. These were categorised as ‘real’ or ‘fake’ through the use of expert fact checkers. In total, 16 published news stories were selected to ensure individual characteristics are controlled for as much as possible. However, to reduce the length of the experiment, the participants were randomly presented with eight of these articles, four of which were real and four of which were fake. After completing the task, the respondents were asked to fill out the questions regarding scepticism and demographics. The titles of the 16 published news stories that were used during the study are presented in Appendix II (Table 2). Whilst during the data collection we used screenshots from the real news stories, in this Table 2 the titles of the articles have been slightly modified and the name of the media outlet that were published in has been omitted.

### Data Collection

In total, 636 social media users participated in our experiment. Out of these, 219 did not complete the experimental tasks and, thus, were removed from the dataset. Out of the remaining responses, 16 did not fill out all questions regarding demographics and scepticism. However, as they did complete the experimental task and their data is sufficient to test H1, their data is retained for this analysis. However, these participants were excluded from the analyses regarding hypotheses set 2 and 3. Out of the remaining 417 respondents, 211 (50.6%) are male, 160 are female (38.4%), and 23 (5.5%) selected the option ‘other.’ Furthermore, the large majority of respondents is between 18 and 34 years old (69.6%). One out of five respondents is older than 34, and 10.5% is younger than 18. Furthermore, a large group of respondents has obtained or is currently obtaining a BSc or MSc degree (57.6%). Finally, the majority of respondents is either working full-time (41.6%) or studying full-time (33.2%).

The data was screened for outliers and other assumptions. To detect outliers, Mahalanobis Distance and Cook’s Distance are used. If a data point exceeds the critical value for both measures, the data is excluded from further analysis. For Mahalanobis Distance, the critical value is set at 18.467, based on Tabachnick and Fidell (2013). The residuals statistics shows us a maximum value of 22.474, thus exceeding the critical value. A closer look at the data shows us that only one data point exceeds this critical value. Given the size of the dataset, a few outliers are not unusual and no immediate cause for concern (Pallant, 2020). However, to ensure this does not cause any issues, Cook’s Distance is considered as well. Following Tabachnick and Fidell (2013), data points with a Cook’s Distance value exceeding 1.0 might cause problems. Looking at the output, we see a maximum Cook’s Distance of 0.051, giving us reason to assume we can continue the analyses using all the data points.

### Measures

Our independent variable (IV) is the training protocol, aiming to find whether it can improve how people score on the dependent variable (DV), which is ‘Ability to detect fake news.’ The variable ‘Training protocol’ is a categorical variable and tells us what condition the participant is assigned to. The variable ‘Ability to detect fake news’ is calculated by assessing how well a participant scored on assessing the news articles. A correct assessment is scored ‘1’ and an incorrect assessment ‘0.’ An overall score was calculated by dividing the total score by eight, which means all scores are between 0 and 1. This variable thus is a ratio variable.

The variable ‘Scepticism’ is added to the conceptual framework as potential mediator. This variable is an ordinal variable and is measured using a scale developed by Obermiller and Spangenberg (1998). The original scale measures the level of scepticism among consumers with regards to advertisements and is adapted to fit the current research. Respondents are asked to answer nine questions on a seven-point Likert scale. The data from these questions is combined into one variable to compute an overall score for ‘Scepticism’ ranging from one to seven, one being the lowest score. This computed variable thus is an interval variable. Finally, demographic factors are included in this research as moderators. There are two variables of interest, namely level of education, and age. The variable ‘Age,’ however, is transformed to a continuous variable when tested as a moderator.

### Data Analysis

For the analysis, first the direct relationship between training protocols and one’s ability to assess misinformation is explored. This allows us to see whether protocols have the potential to train users on recognizing fake news and allows us to explore which protocol works best. For this, an independent samples t-test is used as this test allows for comparison of the scores of multiple groups on some variable, in this case ‘Ability to detect fake news’. Moreover, the direct relationship between ‘Scepticism’ and ‘Ability to detect fake news’ is explored using a correlation analysis. Furthermore, the mediating effect is analysed to see whether ‘Scepticism’ plays a role in explaining the direct relationship between the training protocol and one’s ability to detect fake news.

An independent samples t-test is used to assess the direct relationship between the training protocol and ‘Ability to detect fake news’. First, internal consistency is measured using Cronbach’s *α*. As discussed above, the value for the variable ‘Scepticism’ is calculated using a scale consisting of nine questions. For ‘Scepticism’ Cronbach’s *α* is 0.906 (*M* = 2.53, *SD* = 1.02). This is above the recommended value, and we can thus assume the internal consistency is satisfactory for further analysis. Furthermore, skewness and kurtosis are considered to ensure normality before moving on to the analysis. For skewness, the only variable exceeding 1.000 is gender, implying the distribution of this variable is positively skewed. This can be explained via the descriptive statistics, showing there are more male than female participants. Further tests will show whether the participants have been distributed evenly in terms of gender or whether this skewed distribution has implications. Apart from these tests prior to the analysis, the variable ‘Gender’ will not be used to accept or reject hypotheses. We thus expect no major problems following this skewed distribution. The distribution of age is moderately skewed. In this case, we see a positively skewed distribution, indicating that the distribution leans towards the younger age groups. Looking at the descriptive statistics this makes sense, as the majority of respondents is below 35 years old. Similar to gender, further tests will show whether the age groups were distributed evenly across the conditions and no further action is required at this point. In terms of kurtosis, the variable for most variables falls within the accepted range. For ‘Occupation’ and ‘Gender’, however, the reported values fall outside the range of -1 to 1, indicating that these values are not normally distributed and have a peaked distribution. As these variables are not used for testing hypotheses, no further action is needed. Further tests will show whether the distribution across groups is sufficient. Next, the correlations between the IV are examined to check for multicollinearity (see Table 1).

**Table 1:** Correlation matrix of variables

	**1**	**2**	**3**	**4**	**5**	**6**	**7**	**8**
1. Age	1.000							
2. Gender	0.082 ~	1.000						
3. Level of Education	0.280***	0.058 ~	1.000					
4. Occupation	-0.340***	0.116*	-0.152**	1.000				
5. Fake News Detection	0.089 ~	-0.051	0.153**	-0.089 ~	1.000			
6. Scepticism	-0.213***	0.108*	-0.164**	0.156**	-0.068	1.000		
7. Control Group	0.070	-0.086 ~	0.003	-0.041	-0.172***	-0.019	1.000	
8. Experiment Group	-0.070	0.086 ~	-0.003	0.041	0.172***	0.019	-1.000	1.000

Significance levels: ~ p < 0.1 * p < 0.05 ** p < 0.01 *** p < 0.001

None of the correlations, except for that between the control and experimental group exceeds 0.700, while all Variance Inflation Factor (VIF) values are between 1.000 and 1.300, and do not exceed critical levels. Finally, we test for the normality, linearity, and homoscedasticity of residuals. No major deviations were seen in the Normal P-P plot, suggestion normality. Moreover, the scatterplot has a rectangular shape with no clear pattern, which suggests no violation of the assumptions either. Lastly, no data points fall above or below the 3.3 or -3.3 border, which indicates that there are no outliers that need further consideration (Tabachnick & Fidell, 2013).

### Distribution across conditions and manipulation checks

To ensure that the distribution of participants into groups meets all requirement and all participants are distributed evenly in terms of personal characteristics, such as age and gender, several tests are carried out. First, a Chi-Square Test of Independence is conducted to check the distribution in terms of age, gender, level of education, and occupation. For age, Pearson’s Chi-square value is not significant (χ^2^ (6, *n* = 401) = 10.113, *p* = 0.120, *phi* = 0.159). This indicates random distribution in terms of age groups across the conditions. Pearson’s Chi-square value for gender is not significant either (χ^2^ (3, *n* = 401) = 5.046, *p* = 0.168, *phi* = 0.112), suggesting sufficient random distribution in terms of gender. For level of education, Pearson’s Chi-square value is not significant (χ^2^ (6, *n* = 401) = 3.877, *p* = 0.693, *phi* = 0.098), and for occupation Pearson’s Chi-square value is not significant as well (χ^2^ (6, *n* = 401) = 8.506, *p* = 0.203, *phi* = 0.146). Thus, we can assume that the participants are distributed evenly across the groups and no problems are expected in further steps of the analysis. In addition, a One-Way ANOVA is executed to test the effectiveness of the manipulation. The output shows significant differences in the score on fake news detection between the control group and the experimental group (*F* (1, 415) = 12.647, *p* < 0.005). Therefore, we can assume the manipulation is sufficient and can continue with further data analysis.

### Main effects

To compare the scores on ‘Ability to detect fake news’ between the control and experimental group, an independent samples t-test was conducted. The output shows that the fake news detection score of the control (*M* = 0.78, *SD* = 0.16) and experimental group (*M* = 0.84, *SD* = 0.15) differs significantly (*t* (415) = -3.556, *p* < 0.005), which suggest that the training protocol affects users’ ability to distinguish fake and real news messages.

As the literature gives indications for scepticism, level of education, and age may be related to one’s ability to detect fake news, these are considered as well. Using the correlations from Table 1, we can draw conclusions as to whether a significant relationship exists between these variables. The output shows no direct significant relationship between scepticism and fake news detection (*r* = 0.068, *p* = 0.171). Similarly, a correlation analysis is executed to find whether there is a relationship between age and one’s fake news detection score, with a positive relationship weakly significant at the 90% interval level (*r* = 0.089, *p* = 0.074). We also find a significant positive relationship between level of education and one’s ability to detect fake news (*r* = 0.153, *p* = 0.004).

### Mediation

**Fig. 2:**
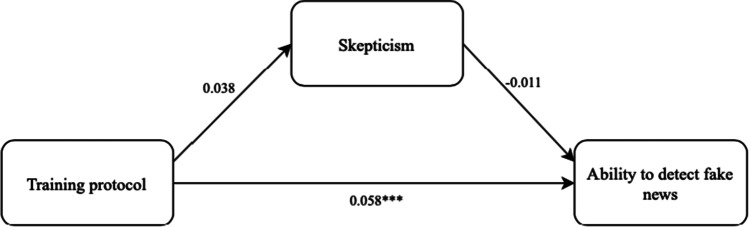
Schematic representation of scepticism as a mediator. Significance levels: ~ p < 0.1 * p < 0.05 ** p < 0.01 *** p < 0.001

After establishing the main effects, the expected mediating effect of ‘Scepticism’ is analysed to find out whether the direct relationship between the training protocol and the public’s ability to detect fake news can be (in part) explained by one’s level of scepticism. For this purpose, the PROCESS macro (Hayes, 2017) is used in SPSS. For this analysis, model 4 with 5000 bootstrap samples is selected and no variables are included as covariates. Furthermore, effects are interpreted as statistically significant in case the confidence interval (CI) does not include 0. Figure 2 presents the results, which show that the training protocol is a significant predictor of ‘Ability to detect fake news' (*b* = 0.058, *SE* = 0.016, 95% CI [0.027, 0.089]). However, looking at the direct relationship between the training protocol and ‘Scepticism,’ no statistically significant effect is found (*b* = 0.038, *SE* = 0.102, 95% CI [-0.163, 0.239]). This indicates that the training protocol does not significantly affect one’s level of scepticism directly. The direct relationship between scepticism and one’s ability to detect fake news was not statistically significant either (*b* = -0.011, *SE* = 0.008, 95% CI [-0.027, 0.004]). This indicates that the respondents’ level of scepticism did not significantly affect their score on fake news detection. The mediation effect is tested using non-parametric bootstrapping. This indirect effect (IE < -0.001, *SE* = 0.002, 95% CI [-0.025, 0.015]) is not significant, and in the current model, no evidence is found for mediation.

### Moderation

**Fig. 3:**
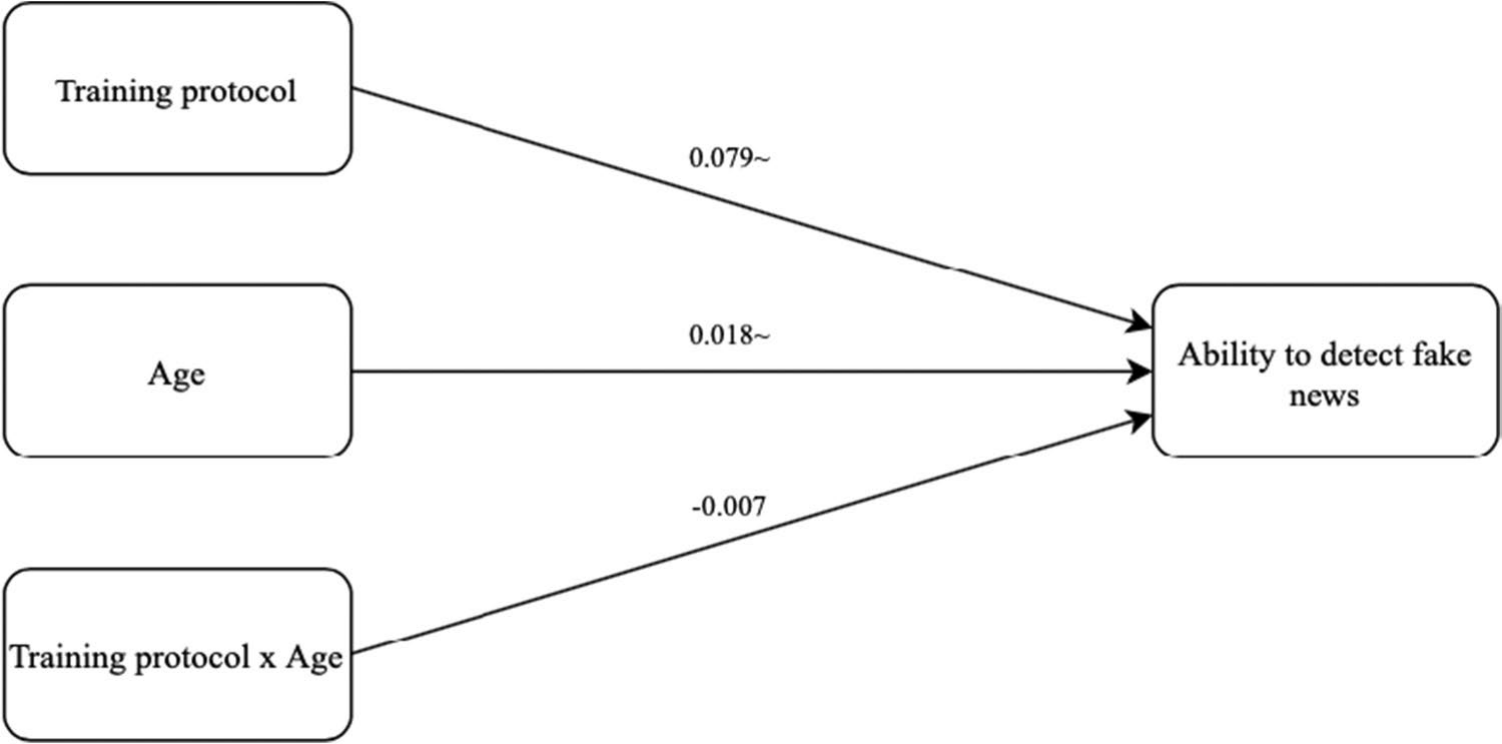
Schematic representation of age as a moderator. Significance levels: ~ p < 0.1 * p < 0.05 ** p < 0.01 *** p < 0.001

To measure the moderation effects, the SPSS PROCESS macro (Hayes, 2017) was used as well. First, the expected moderating effect of age is analysed. For this analysis, the IV is ‘Training protocol’ (coded as 0 = no protocol, 1 = protocol), the moderator variable is ‘Age’, and the DV is ‘Ability to detect fake news’. Model 1 with 5000 bootstrap samples is selected and no variables are included as covariates. Furthermore, effects are interpreted as statistically significant in case the CI does not include 0. Figure 3 presents the results. The output shows that the model as a whole is significant (*F*(3, 397) = 5.686, *p* < 0.001, *R*^2^ = 0.041). The R-squared value suggests that the model can explain 4.1% of the variance in ‘Ability to detect fake news’. While the training protocol (*b* = 0.079, *SE* = 0.042, 95% CI [-0.003, 0.161]) and age (*b* = 0.018, *SE* = 0.100, 95% CI [-0.001, 0.038]) individually weakly predict the participants’ ability to detect fake news, the interaction between these does not significantly predict their ability to detect fake news. Therefore, H3a, in which age was predicted to play a moderating role, is rejected.

**Fig. 4:**
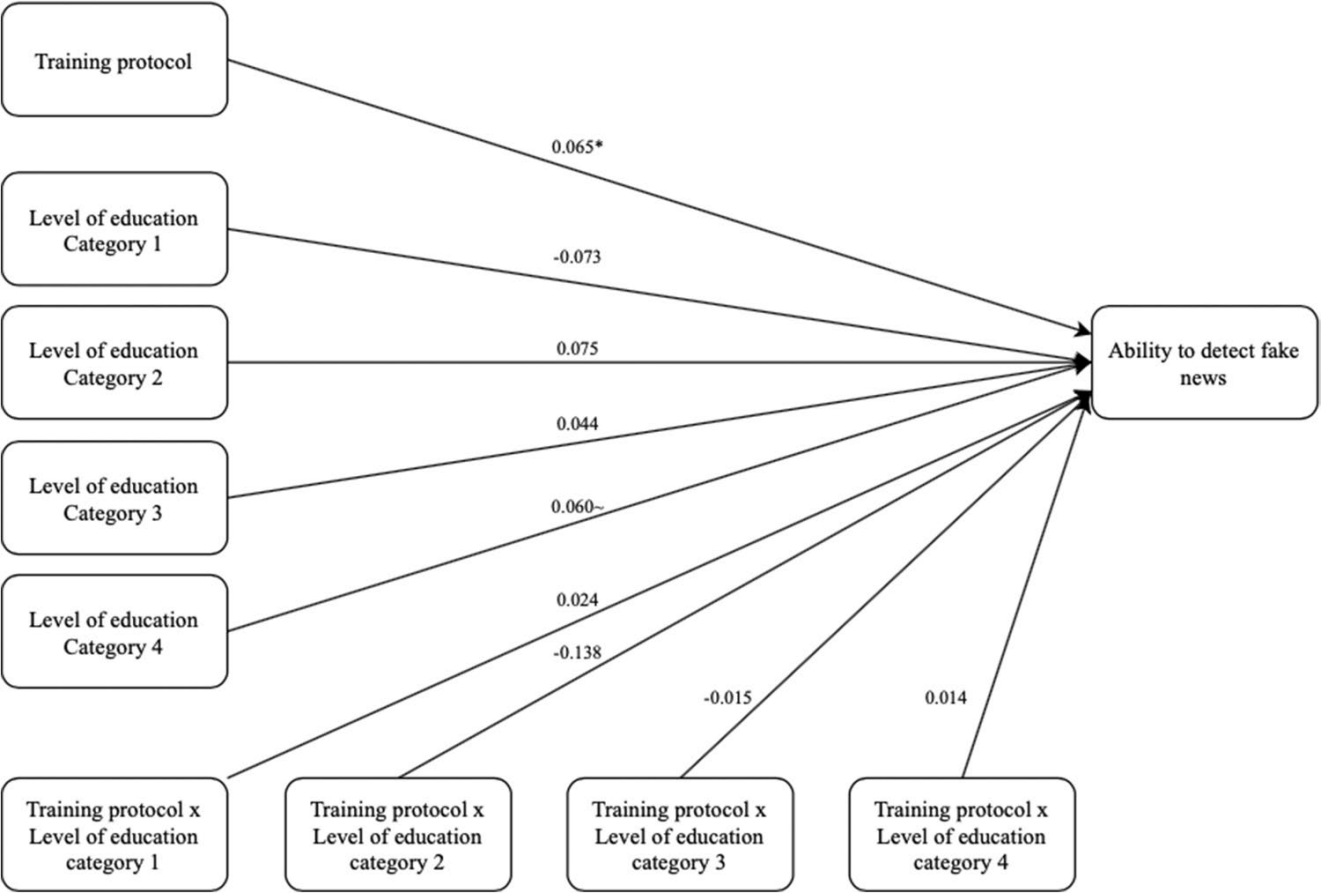
Schematic representation of level of education as a moderator. Significance levels: ~ p < 0.1 * p < 0.05 ** p < 0.01 *** p < 0.001

Second, the moderating effect of ‘Level of education’ is analysed. For this analysis, the IV is ‘Training protocol’ (coded as 0 = no protocol, 1 = protocol), the moderator variable is ‘Level of education’ (coded as 0 = high school, 1 = Secondary vocational education, 2 = Higher professional education, 3 = BSc, 4 = MSc), and the DV is ‘Ability to detect fake news’. Similar to the previous analysis, model 1 with 5000 bootstrap samples is selected and no variables are included as covariates. Furthermore, effects are interpreted as statistically significant in case the CI does not include 0. The output shows that the model as a whole, including all variables, is significant (*F*(9, 352) = 2.761, *p* = 0.004, *R*^2^ = 0.066). The R-squared value suggests that the model can explain 6.6% of the variance in ‘Ability to detect fake news’. Moreover, the output shows that the training protocol significantly predicts ability to detect fake news (*b* = 0.065, *SE* = 0.032, 95% CI [0.001, 0.129]). However, none of the other variables or interactions significantly predict the participants’ ability to detect fake news (see Fig. 4). Thus, H3b, in which a moderation of level of education was predicted, is rejected. Based on the output, we can accept H1. The data show that those participants assigned to the control group score significantly lower on the fake news detection task than those assigned to the experimental group. Based on the results, H2a is rejected. No evidence is found towards a statistically significant relationship between one’s level of scepticism and ability to detect fake news. Similarly, H2b is rejected. Although we expected scepticism to partly explain the relationship between the training protocol and ability to detect fake news, no evidence is found for this mediation. H3a and H3b are also rejected.

We find no evidence for a strengthening or weakening effect of these variables on the link between the training protocol and users’ ability to detect fake news. In addition to clarifying our hypotheses, the results point to two more findings. Although not included in our hypotheses, the results show a weakly significant relationship between age and one’s ability to detect fake news. Conclusions need to be drawn with caution, as the effect is only significant at the 90% interval level, but this finding fits prior research and is considered below. Moreover, the results show a significant relationship between level of education and one’s ability to detect fake news.

## Discussion

Our aim was to explore the role of training protocols in enabling social media users to detect fake news. Prior research suggests using tools on social media and employing fact-checkers, but this direct relationship has not been considered before. Our findings show that those exposed to the training protocol including information on how to recognize fake news score better on fake news detection. Applying this more broadly, this implies that complex education or training tools that require large investments may not be necessary, as relatively simple training protocols consisting of tips and information can lead to favourable results. Scepticism is also considered here, as prior studies suggest that higher levels of scepticism might lead to higher levels of critical analysis of all news stories one encounters. However, we do not find that this enhanced level of critical analysis leads to better performance in detecting fake news. Moreover, scepticism does not act as a mediator and cannot explain the relationship between the training protocol and one’s ability to detect fake news. In addition, demographic variables were also considered. Age seems positively related to one’s ability to detect fake news, implying that the older one gets, the better they are at detecting fake news. While this result needs to be drawn with caution as it is only weakly significant, it confirms findings from prior research stating older generations are better at distinguishing fake news from real ones. Moreover, our findings show a relationship between level of education and ability to detect fake news, suggesting that those that have completed higher levels of education are better at detecting fake news. While a general protocol already reaps positive results, it could be beneficial to tune it to individual needs considering age groups and level of education. The findings regarding age and level of education confirm previous work considering the effect of demographic factors on fake news detection. We expected these variables to play a moderating role—that is, the training protocol would have a stronger positive effect on younger people and on people with lower levels of education. However, the results of the experiment do not provide evidence for this.

### Theoretical Implications

Although the literature discusses many ways to counter fake news and deal with its consequences, for instance using algorithms and fact-checking by experts, there are a lot of limitations to these methods. First, AI is an often-mentioned solution to detecting fake news on social media. However, the development of such AI-based solutions is time-consuming and due to the fast rate at which misinformation technology is developing this is not a comprehensive solution. A second proposed solution is the employment of fact-checkers. However, manual fact-checking is time-consuming and while experts’ knowledge is valuable in this regard, it cannot be a long-term solution. Prior studies, thus, call for educating users and providing them with the tools to individually deal with fake news. We extend this body of literature by developing and studying such a tool aimed at equipping users with the right skills. We address the concerns with existing methods and shows that the protocol can help in countering the effects of fake news and can become a first step in the development of effective training protocols.

Our work has a clear focus on the extant Information Systems research agenda (Struijk et al., 2022) and its theoretical implications can go beyond the topic of misinformation on social media. For instance, the training protocols that we propose in this study can be enhanced by a gamification element (e.g., Alexiou et al., 2021) to better address the public and especially the younger generations. To this end, our work is a starting point for research focusing on how such training protocols should be designed and how different groups of people can be targeted most efficiently; for instance, based on age or level of education. The relationship between level of education and one’s ability to detect fake news asks for more insight into where these difference in score comes from and how this can be tackled. Future research should further consider this in the development of such training protocols, along with how each of these groups can be reached and trained in the best way possible.

### Practical Implications

The consequences of fake news are increasingly harmful in a time when we rely on social media. Filter bubbles and echo chambers lead to users being exposed to information that fits their existing beliefs. This has negative consequences, regardless of whether the stories are real or fake. The effect of fake news combined with effects of filter bubbles and echo chambers may increase polarization through a lack of exposure to balanced news. We answer calls in the literature on empowering and educating users about fake news and their consequences and propose a new solution that makes use of fact-checkers’ knowledge and simultaneously is easily scalable without requiring large investments. Our solution is not proposed as a substitute for the above-mentioned solutions, but rather as a complementary tool, which is suitable for use on social media platforms, such as Facebook, and on news outlets’ websites. These organisations can use such a tool to ensure higher quality content, raise awareness among users, and educate them about how to evaluate news. This can help the public increase their skills to critically analyse news and may in turn help in addressing more recent concerns about the declining trust in traditional news outlets. In this way, experts’ knowledge can be shared with the public to help them improve their skills and become better at detecting fake news. Although most suited to social media platforms and news outlets, the protocol is potentially suited for broader use as well, for instance on websites and platforms owned by the government. This allows for broader spread of and attention to information regarding fake news. Moreover, widespread use of this tool can encourage awareness and lead to more critical analysis of all information presented. Furthermore, as education seems to play a significant role, we suggest more education on fake news and its negative consequences, especially during earlier stages of education is worth looking into, as this likely helps in reducing these differences later in life. We therefore suggest the use of these training protocols in schools.

### Limitations and Future Research

Although we followed a structured design, our work has limitations that need to be acknowledged. First, the experiment is carried out online, which although does not have direct implications for the results, it implies a lack of control. Moreover, the participants are from around the world, while the news articles presented during the data collection are in English. Differences in culture and native language may affect the way in which participants assess the news articles and answer the questions, as knowledge about local politics and other developments might play a role. Prior research suggests the potential effect of culture (Rampersad & Althiyabi, 2020), however, as no questions regarding nationality, language, or culture were asked, no conclusions can be drawn in this regard. Future research could consider this and investigate different versions of the protocol that might be suited to certain groups of people based on demographics. Moreover, the news stories for the experiment are derived from eight news outlets that are selected based on how they are evaluated by fact-checking websites. However, the news theme is not included as a selection criterion. As emotion plays a large role in fake news (Harber & Cohen, 2005), broader themes might affect how one evaluates a story. Future research could consider how themes, for instance health, climate change, or politics, affect how participants interpret news stories. Furthermore, based on prior literature, there is reason to believe one’s existing skills might play a role as well. That is, those with lower initial skills will benefit from such protocols to a greater extent than those already well-equipped to distinguish real and fake stories. As we established that the training protocol can play a positive role, future research could look on how protocols can be adjusted to benefit these groups as efficiently as possible. Finally, the existing body of literature considers satire as well (Rubin et al., 2016). Although the objective of satire in many cases is different than that of fake news and misinformation—these messages are often humorous—the results can be negative as well, for instance because these messages can affect popular opinion about (groups of) people. Future research could also consider how protocols may help the audience to detect satire as well. Finally, as misinformation has a clear pathway from online to offline social networks, future research should explore how such networks emerge and evolve over time (e.g., Angelopoulos & Merali, 2017), and how the organizations behind popular social media platforms can help in reducing the problem both in online and offline settings (e.g., Angelopoulos et al., 2021).

## Conclusion

We explore the effect that training protocols can have on social media users’ ability to detect fake news. Such training protocols can assist in countering the negative effects of fake news and misinformation and our findings can be seen as a bedrock for further research into how these protocols should be shaped and how they can be tuned to the needs of different groups. Our study is the first to report the potential effect of training protocols on the ability to detect fake news. Our conclusions were drawn based on data collected using an online experiment in which more than 400 participants were asked to complete a fake news detection task. Additional variables were taken into consideration to see whether other factors might play a role. The role of age and level of education indicate that these demographic factors should be considered in the battle against fake news and misinformation. We established the positive effect training protocols have and give way to further research into the development and employment of these protocols. Moreover, we show that employment of such protocols by social media and news organizations might help in educating users and encouraging them to seek a more balanced set of news.

## Appendix I

### Questionnaire

**Part 1. Introduction**

**Table:**

Welcome to this survey
*Please note: It is easier to fill out this survey on a computer, but a mobile device works as well* Thank you for taking the time to participate in this survey. [Details about researchers and institution] Filling out this survey will take approximately 5–10 min. Please remember that there are no wrong answers and that your responses will be treated in a confidential manner. The data collected will only be used for the study and will not be shared with third parties If you are interested in the results of this study, please let us know by sending us an e-mail. We are also available to answer any questions you might have regarding this study. You can reach us via [email] Please click on the arrow below to start the questionnaire

**Part 2. Intervention**

*Group 1.*

Group 1 will not be presented with the protocol and serves as the control group. They will be presented with a general instruction for the task that follows.

**Table:**

Please read through the following text carefully
In the following section, you will be presented with several news articles. For each article, please indicate whether you think the message is real or fake. You can do so by selecting one of the buttons presented below the image. It is not necessary to read through each article in detail Please note: in case you are completing this survey on a mobile device, you can zoom in on the images

*Group 2.*

**Table:**

Please read through the following text carefully
Over the past years, the use of social media has risen and apart from connecting with friends, we increasingly use Facebook and other platforms to access news messages. Although traditional news outlets use social media to share their news stories, these platforms are used for the spread of misinformation and fake news as well—in some cases it can be hard to distinguish these real stories from fake news There are some basic things you can take into account to assess whether stories are real. Please read through the following tips carefully 1. Use your intuition. Ask yourself questions: does the source seem trustworthy? Does the news message look authentic and truthful? You can, for instance, also look at the news outlet's logo and the website as a whole. If your intuition tells you the news message does not seem credible, chances are you are looking at fake news 2. Check the URL. Often, you will recognize large news corporations by merely looking at the URL (the link to the website presented in the search bar on top of the browser). Does something in the URL seem odd? You could be visiting a website that tries to imitate another website 3. Evaluate the content. Look at the headline and the general content of the article. Check the supporting resources used to support the claims. Does it seem like the writer of the article used sound sources? 4. Examine the sentiment. Often, fake news messages are about controversial messages or try to appeal to your emotions, for instance using fear. If you see this tactic, this could be a strong indicator that the message is fake In the following section, you will be presented with several news articles. For each article, please indicate whether you think the message is real or fake. You can do so by selecting one of the buttons presented below the image. It is not necessary to read through each article in detail *Click on the arrow below to continue*

**Part 3. Assessing news messages**

The respondents are presented with news messages and asked to indicate whether they think the message is real or fake. Half of the messages presented are fake. Out of sixteen messages, each participant randomly sees eight.

**Part 4. Scepticism**

To assess the expected mediating impact of scepticism, nine questions are asked, based on a previously developed scale. Answer options ranged from ‘strongly disagree’ to ‘strongly agree on a seven-point Likert scale.

**Table:**

1. We can depend on getting the truth in most news messages on social media 2. Social media news messages’ aim is to inform citizens 3. I believe news messages on social media are informative 4. News messages on social media are generally truthful
5. News messages on social media are a reliable source of information 6. News messages on social media are truth well told 7. In general, news messages on social media present a true picture 8. I feel like I’ve been accurately informed after reading news messages on social media 9. Most news messages on social media provide citizens with essential information

**Part 5. Demographics**

Finally, the respondents are presented with questions regarding demographics.

**Table:**

Finally, I would like to ask you some concluding questions. Please remember that your answers will be treated confidentially, will not be used for any purpose other than this research, and that any information you share cannot be traced back to you
How old are you? ○ < 18 years old ○ 18 – 24 years old ○ 25 – 34 years old ○ 35 – 44 years old ○ 45 – 54 years old ○ 55 – 64 years old ○ > 65 years old What is your gender? ○ Male ○ Female ○ Other ○ I prefer not to say What is your highest level of education (current or obtained)? ○ High school ○ Secondary Vocational Education (MBO) ○ Higher professional education (HBO) ○ University Bachelor ○ University Master ○ I prefer not to say Which of the following best describes your current situation? ○ Working fulltime (≥ 36 h) ○ Working part-time (< 36 h) ○ Retired ○ A full-time student ○ Unemployed ○ Other: I prefer not to say

**Part 6. Debrief**

## Appendix B. News messages

News messages presented during the experiment

Fake news messages

**Table:**

You've reached the end of this survey. Thank you for your time
You just participated in an experiment regarding fake news and misinformation. During the experiment, you were randomly assigned to one of two groups. The first group merely got to see a general description of the task at hand, while the second group got a more extensive description including tips on how to identify fake news. The aim of the study is to find out whether such 'training protocols' may be helpful in improving our skills to detect fake news Thank you for your time. If you have any questions, please feel free to contact us: [email address]

## Appendix II

**Table 2:** List of articles used during data collection

#Page	Title	Fact Checked
1	Study shows more babies killed in abortions due to covid financial problems	*Fake*
2	Biden threatens to cancel 4th of July if Americans don't get vaccinated	*Fake*
3	CDC reports that thousands of Americans have contracted covid after being fully vaccinated, many have died	*Fake*
4	Undercover video proves that CNN is covering up more disturbing crimes	*Fake*
5	Immunologist: Covid vaccines could cause long-term chronic illness	*Fake*
6	Illegal border crossing surge by 400% in Maine	*Fake*
7	These are the problems women may face after getting a covid vaccine	*Fake*
8	Trump is trying to get Mike Pence impeached	*Fake*
9	Why are Australian officials hinting at war with China?	*True*
10	New Jersey offers free beer to residents who get vaccinated in May	*True*
11	Brother of Honduran president sentenced to life in drug case	*True*
12	At military camps in the Myanmar jungle, doctors and students learn how to fire guns	*True*
13	Night-time modes on smartphones don't help with sleep, new research suggests	*True*
14	Case asking courts to free elephant "imprisoned" in Bronx zoo heads to New York's highest court	*True*
15	Trillions of cicadas about to emerge across US	*True*
16	Most of US experienced warming trend over last 30 yeas: NOAA	*True*

Note: Whilst during the data collection we used screenshots from the real media outlet, in this table the titles of the articles have been slightly modified and the name of the outlet has been omitted.
